# How to Apply Predictive Model in Real‐World Practice: The Standard of Model Validation

**DOI:** 10.1002/clc.70096

**Published:** 2025-02-14

**Authors:** Rungroj Krittayaphong

**Affiliations:** ^1^ Department of Medicine Faculty of Medicine Siriraj Hospital, Division of Cardiology, Mahidol University Bangkok Thailand

**Keywords:** anticoagulants, atrial fibrillation, intracranial hemorrhage, stroke prevention

We would like to thank Naoya Kataoka and Teruhiko Imamura for their comments regarding the issues that might be of concern. We would like to respond to clarify comments.

There might be a question about the justification for comparing the COOL‐AF predictive model with the HAS‐BLED model for intracranial hemorrhage (ICH), as the variables of the two models are different. HAS‐BLED was designed to predict major bleeding [1] whereas our predictive model is focused on ICH [2]. We would like to explain the rationale for using ICH as the primary outcome of our study. ICH was chosen as the main outcome for several reasons. First, a previous study showed that Asian patients with atrial fibrillation (AF) who used warfarin had a fourfold increased risk of ICH compared to non‐Asians [3] and the results of the four DOAC trials demonstrated a much higher rate of ICH in Asians compared to non‐Asians [4]. Second, among the 3405 patients in our registry, 199 (5.5%) developed major bleeding during follow‐up, with 70 cases of ICH (36% of all major bleeding). Moreover, the mortality rate from ICH was 39% compared to 14% of non‐ICH major bleeding and 15% for ischemic stroke. Therefore, we chose ICH as the main outcome and the primary target for developing the prediction model.

We performed additional analysis to determine whether each component of the HAS‐BLED score is a significant predictor for either ICH or major bleeding in the population of our study. The components of the HAS‐BLED score are as follows: uncontrolled **H**ypertension, **A**bnormal renal, or liver function; history of **S**troke; history of **B**leeding; **L**abile international normalized ratio (INR); **E**lderly (age above 65 years); and, **D**rugs or alcohol (1 point each). We identify that age > 65 years, labile INR, and abnormal liver function are predictors of ICH, while only age > 65 years is a predictor for major bleeding. Therefore, the score developed from one population may not be applicable or suitable for another population. It is important that we use our own data from our own population. When we want to apply the predictive model from another study to our population, we must carefully consider the basis of the predictive model and decide whether the nature of its development and validation is suitable for the population of interest.

The predictive model of our study was developed following the standard procedure outlines in the Prediction model Risk Of Bias ASsessment Tool (PROBAST) [5]. We performed C‐ and d‐statistics using Bootstrap for internal validation. We used the Brier score to assess the predictive ability of the model. Additionally, the C‐statistics, calibration slope and intercept were corrected for the optimism. We complied with the guidance of the Transparent Reporting of a Multivariable Prediction Model for Individual Prognosis or Diagnosis (TRIPOD) reporting guideline [6].

Regarding the comments on the predictive model for ischemic stroke/systemic embolism (SSE) and heart failure in the COOL‐AF registry, we have previously reported a separate model for SSE and heart failure in earlier publications [7, 8]. The variables in the prediction model of SSE included hypertension, chronic kidney disease and oral anticoagulants (OAC), while the variables for heart failure included age, female sex, history of heart failure, history of coronary artery disease, cardiac implantable electronic device, diabetes, hypertension, smoking, renal replacement therapy (RRT), and LVEF < 50%.

Regarding the low proportion of direct oral anticoagulants (DOAC), we have already mentioned this point in the limitations of study. This is due to the registry being conducted during 2014–2017 the time when DOACs were not widely used, and also due to the reimbursement issue. We also addressed the limited generalizability of the model's application. However, our models have been tested in the Asia‐Pacific Heart Rhythm Society (APHRS) cohort [9].

We appreciate the comment regarding the removal of patients with RRT, as this group may not be suitable for OAC. We re‐analyzed the data after excluding 40 patients with RRT. The results showed that the four variables remained in the final prediction model for ICH, namely: age, female sex, nonsmoking, and OAC. The C‐statistic of the model after excluding patients with RRT was 0.707 (0.691–0.723) which is not significantly different from the original model [0.717 (0.702–0.732)].

## Conflicts of Interest

The author declares no conflicts of interest.

## Data Availability

The dataset that was used to support the results and conclusion of this study are included within the manuscript. The additional data are available from corresponding author upon reasonable request.
